# A Medium-Throughput System for In Vitro Oxidative Stress Assessment in IPEC-J2 Cells

**DOI:** 10.3390/ijms21197263

**Published:** 2020-10-01

**Authors:** Miriam Ayuso, Steven Van Cruchten, Chris Van Ginneken

**Affiliations:** Laboratory of Applied Veterinary Morphology, Department of Veterinary Sciences, Faculty of Biomedical, Pharmaceutical and Veterinary Sciences, University of Antwerp, 2610 Wilrijk, Belgium; Steven.vancruchten@uantwerpen.be (S.V.C.); Chris.vanginneken@uantwerpen.be (C.V.G.)

**Keywords:** ROS, oxidative stress, in vitro, IPEC-J2, antioxidants, menadione, fluorescence

## Abstract

The feed industry continuously seeks new molecules with antioxidant capacity since oxidative stress plays a key role in intestinal health. To improve screening of new antioxidants, this study aims to set up an assay to assess oxidative stress in the porcine small intestinal epithelial cell line IPEC-J2 using plate-reader-based analysis of fluorescence. Two oxidants, H_2_O_2_ and menadione, were tested at 1, 2 and 3 mM and 100, 200 and 300 µM, respectively. Trolox (2 mM) was used as the reference antioxidant and the probe CM-H2DCFDA was used to indicate intracellular oxidative stress. Cell culture, reactive oxygen species (ROS) production and assessment conditions were optimized to detect a significant ROS accumulation that could be counteracted by pre-incubation with trolox. Menadione (200 µM) reproducibly increased ROS levels, H_2_O_2_ failed to do so. Trolox significantly decreased intracellular ROS levels in menadione (200 µM)-exposed cells in a consistent way. The system was further used to screen different concentrations of the commercially available antioxidant ELIFE^®^. Concentrations between 100 and 200 ppm protected best against intracellular ROS accumulation. In conclusion, the combination of CM-H2DCFDA fluorescence analysis by a plate-reader, trolox as a reference antioxidant and 200 µM of menadione as a stressor agent, provides a replicable and reliable medium-throughput setup for the evaluation of intracellular oxidative stress in IPEC-J2 cells.

## 1. Introduction

**Figure 1:**
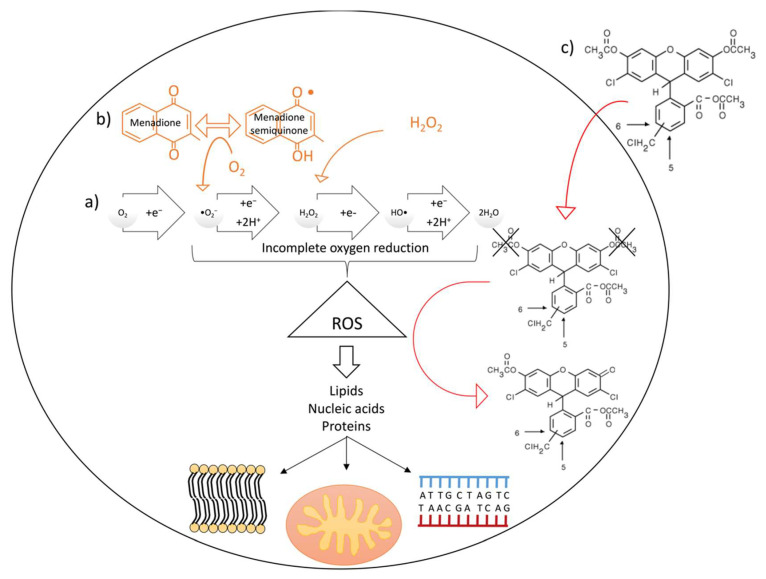
(**a**) Naturally occurring intracellular reactive oxygen species (ROS) production and consequences on cellular components (cell membranes, mitochondria and DNA); (**b**) H_2_O_2_ and menadione contribution to intracellular ROS accumulation (orange arrows indicate how these molecules contribute to the incomplete reduction of oxygen) and (**c**) CM-H_2_DCFDA ROS-sensitive probe mechanism of action. Red arrows indicate movement of the probe from the extracellular to the intracellular space (up) and contribution of ROS to the oxidation of the probe (down).

Reactive oxygen species (ROS) such as superoxide anion (•O^2−^), hydrogen peroxide (H_2_O_2_), and hydroxyl radical (HO•), are byproducts of normal cellular metabolism formed upon partial reduction of O_2_ (Figure 1a). Reactive oxygen species function as second messengers [1] and have beneficial effects on several processes [2]. In healthy conditions, the formation of excessive levels of oxidants and the associated oxidative stress is counterbalanced by the presence of a variety of antioxidants. 

The gastrointestinal epithelium is particularly exposed to ROS, due to its contact with the external environment, including dietary factors, and the intestinal flora, which are potential sources of ROS. Moreover, these xenobiotics can activate intestinal immune cells to produce inflammatory cytokines and other mediators that exacerbate oxidative stress [2]. Although ROS play a role in host defense due to its bactericidal properties [3], excessive levels of ROS negatively affect cell viability via lipid peroxidation and protein and nucleic acid oxidation (Figure 1). As such, ROS damages cytoskeletal proteins, tight junctions [4], alters intestinal cells mitosis, apoptosis and differentiation [5,6], all resulting in cell and tissue impairment [7]. As a consequence, ROS are linked to several gastrointestinal tract (GIT) disorders [8]. Due to the importance of a proper redox status for (intestinal) health in humans and other species, there is a continuous interest in the effects of antioxidants on cellular ROS metabolism [9,10,11,12,13,14]. In this respect, an in vitro approach is often used to study ROS metabolism and to screen, in a preliminary step, compounds or combinations of compounds with a potential antioxidant capacity. These in vitro setups mimic, to a large extent, the physiology and molecular mechanisms observed in living animals and humans [15,16]. Additionally, their use has a lower cost, shorter operation time and a lack of ethical concerns when compared to in vivo screenings [17,18,19]. Nevertheless, some differences exist between in vivo and in vitro cell response to oxidative stress, which depends on the adaptation of the cell line to the growth culture media or to the contribution of the latter to the overall oxidative stress in the culture [20]. 

Several cell lines have been used in oxidative stress research. Human cells, such as HepG2, L-929 or erythrocytes [21,22,23] were popular in the early 2000s. More recently, the intestinal Caco-2 cell line was proposed as a better candidate for in vitro antioxidant activity assays [19]. This cell line overcomes the main limitation of the aforementioned non-intestinal cell lines, namely their lack of absorptive capacity. Human Caco-2 cells were reported to effectively predict in vivo intestinal absorption and drug bioavailability in humans [24,25]. In view of antioxidant activity assays this feature is highly relevant since antioxidants are mostly taken orally—in either foods or food supplements—and need to be absorbed by intestinal cells in order to exert an intracellular and a systemic effect. However, Caco-2 cells are a cancer-derived cell line that has limitations in translation to in vivo intestinal cells because of an altered glycosylation pattern, aberrant protein expression and unresponsiveness to hormones or cytokines [26]. Therefore, the use of non-cancerous cell lines derived from rodent (IEL) or pig small intestine (IP, IPEC-1 and IPI-21) was introduced. The interest in using in vitro systems derived from pig intestinal cells lies in the importance of GIT health in pigs used in meat production, and in the usefulness of this species as a model for human GIT [27,28]. Among the lines derived from the pig, IPEC-J2 cells are the most frequently used [17,29,30,31]. This cell line presents several advantages over others, namely its morphological similarity with intestinal epithelial cells in vivo, the expression of tight junction proteins and the synthesis of cytokines, defensins, toll-like receptors and mucins (reviewed in [17,29,30]. Previous work has demonstrated the usefulness of an IPEC-J2 cells-based model in the study of the antioxidant capacity of several compounds prior to their in vivo validation in piglets [18]. In this model, the antioxidant capacity of a compound is determined by measuring its scavenging potential upon incubation of cells with an oxidant by using a fluorescent probe that allows for the intracellular quantification of ROS levels [17,18]. 

To evaluate the scavenging capacity of tested compounds, cells must be stressed to increase intracellular ROS production. To this end, several molecules can be used: H_2_O_2_ is commonly used as a stressor agent. It is naturally synthesized by cells and plays a physiological role as a signaling molecule [32,33,34] but its excess leads to cell damage. Other molecules such as menadione or diethyl maleate can also increase cellular oxidative stress. Menadione, also known as vitamin K3, is a cytotoxic drug used as a chemotherapeutic agent [35] that produces large amounts of superoxide anion intracellularly (Figure 1b) [36]. It has been previously used to induce oxidative stress in various systems, including cell culture, vertebrate embryos and fungi [37,38,39,40,41]. Once cells have been stressed, the scavenging ability of different antioxidants is frequently compared to the potential of trolox ((±)-6-hydroxy-2,5,7,8-tetramethylchromane-2-carboxylic acid), considered as the reference antioxidant in cell culture-based antioxidant studies [17]. Trolox is also used in the 6-hydroxy-2,5,7,8-tetramethyl chromane-2-carboxylic acid equivalent antioxidant assay (TEAC) to assess the antioxidant capacity of compounds, both in vitro [42,43] and in biological samples, such as the plasma of animals subjected to antioxidant supplementation [44]. One of the most commonly used ROS-reactive fluorescent probes is 2′,7′-dichlorodihydrofluorescein diacetate (H_2_DCFDA). However, this probe has a high passive leakage across the plasma membrane [45]. To overcome this problem, a variant of the probe that displays a lower passive leakage from the cell was developed [46]. In this new probe, chloromethyl groups are added in the molecule (CM-H_2_DCFDA). When this probe is oxidized (CM-DCFDA) by the intracellular presence of ROS (Figure 1c), it is reported to present negligible leakage out of the cell [46] and is therefore considered a good intracellular ROS accumulation marker. Subsequently, ROS production can be calculated from fluorescence values measured in several thousands of individual cells by flow cytometry [17,18]. This procedure, although highly sensitive, specific and powerful, has some limitations such as long analysis times, high costs and a huge amount of data, which can be difficult to analyze. Alternatively, ROS accumulation-derived fluorescence can been measured in a microplate reader [47], a relatively accessible and inexpensive piece of equipment that allows the simultaneous assessment of many samples. However, a proper protocol for general use is lacking.

Thus, the aim of this work was to develop a method for fast, medium-throughput in vitro oxidative stress evaluation in IPEC-J2 cells based on microplate reader-fluorescence analysis. This alternative method should allow us to investigate the effects of oxidative stress on intracellular ROS accumulation in IPEC-J2 cells. To develop this method, the culture and test conditions (culture plate, reading conditions, oxidant concentration, antioxidant effect, the detection probe) were optimized to permit the detection of: (a) a significant rise in the intracellular ROS levels that (b) can be prevented by the use of trolox as reference antioxidant. Once the optimal conditions were determined, we validated this system with a commercially available natural antioxidant (ELIFE^®^).

In spite of the high variability observed in our system, the combination of CM-H2DCFDA fluorescence analysis by a plate-reader, trolox as a reference antioxidant and 200 μM of menadione as a stressor agent, provided a replicable and reliable setup for the evaluation of intracellular oxidative stress in IPEC-J2 cells. Moreover, ELIFE^®^ at concentrations between 500 and 1000 ppm was effective at ameliorating the rise in ROS accumulation produced by menadione.

## 2. Results

### 2.1. Imaging Plate Selection for Cell Culture

**Figure 2:**
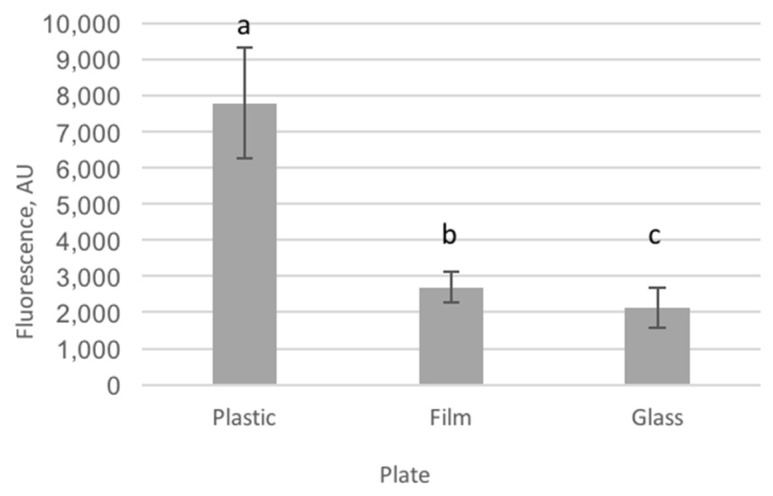
Autofluorescence of the three tested cell culture plates, differing in the material used in the bottom (plastic: polystyrene cell culture microplate Clear^®^ (Greiner Bio-One, Vilvoorde, Belgium); film: cell imaging microplate 25-μm film bottom (Eppendorf, Aarschot, Belgium) and glass: cell imaging microplate 170-μm coverglass bottom (Eppendorf)). Fluorescence intensity is represented in arbitrary units (AU). Plastic-bottomed plates show the highest autofluorescence values, while glass-bottomed plates showed the lowest. Film-bottomed plates showed intermediate autofluorescence that statistically differed from the two former plate types. Different letters denote values that are significantly different.

Background fluorescence was measured in plastic, film and glass plates to select the plate showing the lowest autofluorescence. Plastic plates showed the highest autofluorescence and glass plates the lowest (*p* < 0.001, Figure 2). Film plates showed lower (3-fold) autofluorescence than plastic but slightly higher (1.3-fold) autofluorescence than glass plates. 

**Figure 3:**
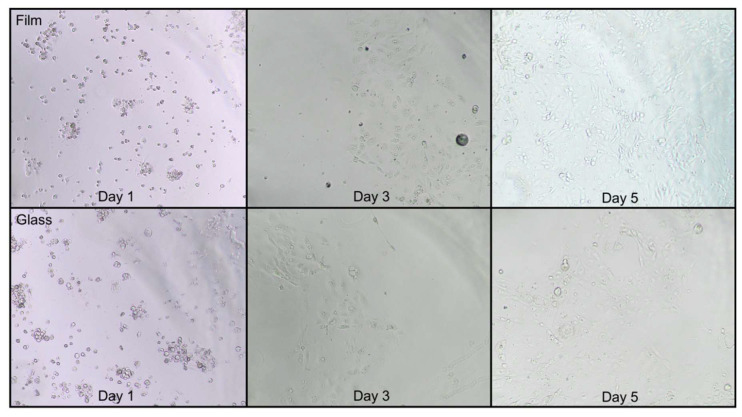
Cell growth in film and glass-bottom plates in days 1, 3 and 5 after seeding. Cell growth was better and convergence was attained in film- but not in glass-bottom plates at day 5 (10×).

In Figure 3, a clear difference in cell growth is observed between film and glass plates. In the film plates, confluence was nearly complete at day 5 but not in glass plates. 

### 2.2. Intracellular Oxidative Stress

#### 2.2.1. Plate Reading Conditions

Fluorescence was read in full (before discarding growth medium) and empty (after discarding growth medium and washing cells with PBS for three times) wells. Full wells provided data to which the statistical model adjusted better, since they showed a lower Akaike information criterion (AIC), independently of the stressor or concentration used (AIC was −127.7 and −97.0 in full menadione and H_2_O_2_-stressed wells and −40.7 and 37.4 in empty menadione and H_2_O_2_-stressed wells, respectively). On the other hand, the within-group coefficient of variation (CV) was statistically lower in full when compared to empty wells (0.88 and 2.7, respectively; *p*-value = 0.029).

#### 2.2.2. Experimental Setup

At the start, the setup previously used by Vergauwen and co-workers was followed. However, very low fluorescence values were obtained when using a plate-reader-based fluorescence assessment (data not shown). Thus, a modification of this method was performed (the fluorescence probe was incubated after cells were exposed to the stressors), leading to higher fluorescence levels that could be quantified by the plate reader.

#### 2.2.3. Oxidative Stress Assessment

Five concentrations were screened for H_2_O_2_: 1, 1.5, 2, 2.5 and 3 mM H_2_O_2._ After preliminary tests showed an excessive cell death induced by incubation with 300 μM menadione, menadione was tested at only four different concentrations: 100, 150, 200 and 250 μM menadione. 

Three independent experiments (Ex1, Ex2 and Ex3) were performed in a period of 6 months. Each experiment consisted of three independent replicates, which were run in different weeks. The results below include those obtained in each experiment separately and after pooling the data coming from the three different experiments (for more information on the analysis, see statistics section).

**Figure 4:**
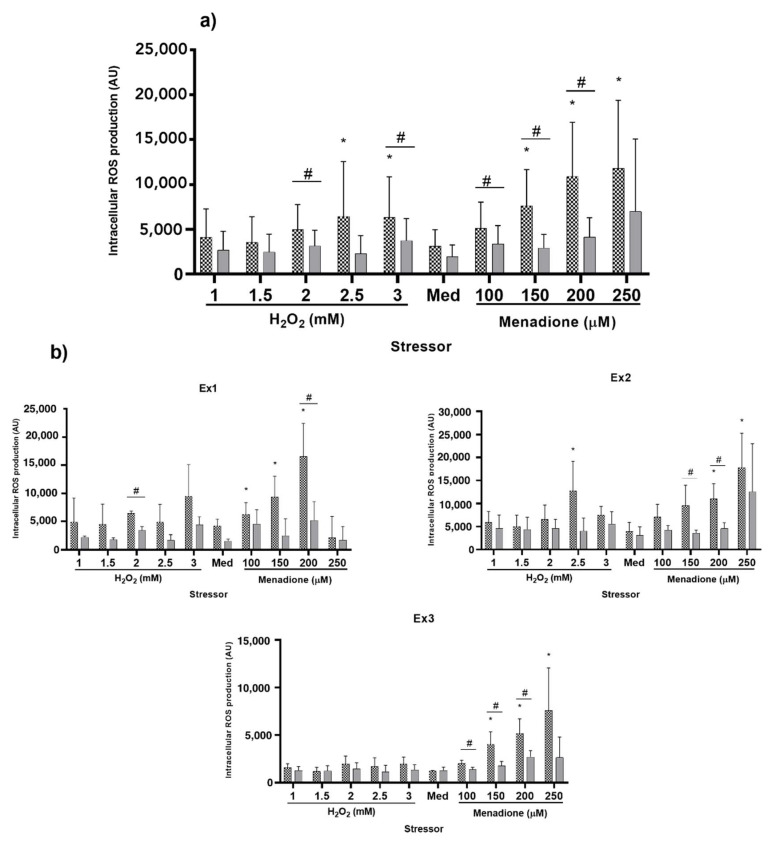
ROS production after an 18-h pre-incubation with: control (colorless growth medium containing no antioxidant, dotted bars) and trolox (colorless growth medium containing 2 mM trolox, solid grey bars), followed by a one-hour incubation with medium (Med; colorless growth medium containing no stressors) or different stressors (colorless growth medium containing either H_2_O_2_ or menadione at the specified concentrations). ROS production in different conditions was determined by fluorescence measurement (measured in arbitrary units, AU). Different asterisks (*) indicate statistically significant differences in ROS production in stressed vs. non-stressed (medium) cells (i.e., stressor effect). Hashtags (#) represent a statistically significant decrease in ROS production after trolox incubation when compared to the same stressor treatments in control cells (antioxidant effect). *p* < 0.05. (**a**): Pooled data obtained in three independent experiments performed in a lapse of 6 months. (**b**): Individual data of each experiment (Ex1, Ex2 and Ex3) consisting of three independent replicates. Data represent means ± SD (*n* = 9 in (**a**) and *n* = 3 in (**b**)).

First, the stressor capacity of the different agents was evaluated. When using the whole set of data (*n* = 9), H_2_O_2_ at 2.5 and 3 mM as well as menadione at 150 to 250 μM showed a statistically significant increase in ROS production when compared to non-stressed cells (*p* < 0.0001 for the stressor effect; Figure 4a). The stressor effect was observed in all three experiments (*p* = 0.0322 in Ex1, *p* = 0.0026 in Ex2 and *p* < 0.0001 in Ex3). Only 200 μM menadione was identified to increase intracellular ROS accumulation in all three independent replicates. Moreover, menadione at a concentration of 150 and 250 μM significantly increased intracellular ROS in two out of three experiments (Figure 4b).

Second, we assessed the efficacy of the reference antioxidant (2 mM trolox) to decrease intracellular ROS in non-stressed cells and cells stressed with the different oxidants at different concentrations (Figure 4a,b). Trolox significantly decreased the production of intracellular ROS in cells incubated with 2 mM H_2_O_2_ (37%; *p* = 0.0257), 3 mM H_2_O_2_ (41%; *p* = 0.0197), 100 μM menadione (38%; *p* = 0.0140), 150 μM menadione (61%; *p* = 0.0009) and 200 μM menadione (62%; *p* = 0.0001). Regarding the results obtained in each individual experiment, the results for 200 μM menadione were consistent in all three replicates while trolox protected the cells from the stressor effects of 150 μM menadione in two out of the three experiments (Figure 4a,b). Thus, 200 μM menadione meets the established criteria for stressor selection: it produces a significant rise in the intracellular ROS levels that is prevented by the use of trolox in a consistent way throughout time and with as little as three replicates per experiment.

However, the system showed high variability. Within each experiment (*n* = 3), the coefficient of variation (CV) for the three replicates varied from 37 (Ex1) to 47% (Exp2) with an average CV of 42%. Moreover, the variation was slightly higher in cells that were pre-treated with trolox than with medium (40 vs. 45%). Regarding the stressors, menadione at 100 and 150 μM produced the lowest variability (24 and 31%, respectively). The average CV for non-stressed cells was 38%, whereas increasing the concentration of menadione up to 200 μM led to a CV of 35%.

Higher variation was observed when data from all three experiments were combined, with the average CV for all conditions as high as 73%. All CVs for the different conditions and data sets can be found in the Supplementary Table S1.

#### 2.2.4. Validation of the System with ELIFE

Eight different doses of a natural antioxidant (ELIFE^®^) were tested to determine the optimal dose for oxidative stress protection in IPEC-J2 cells. In preliminary tests, high doses of ELIFE^®^ (1250 and 1500 ppm) were observed to drastically reduce cell viability. Thus, these two concentrations were excluded in further experiments. 

**Figure 5:**
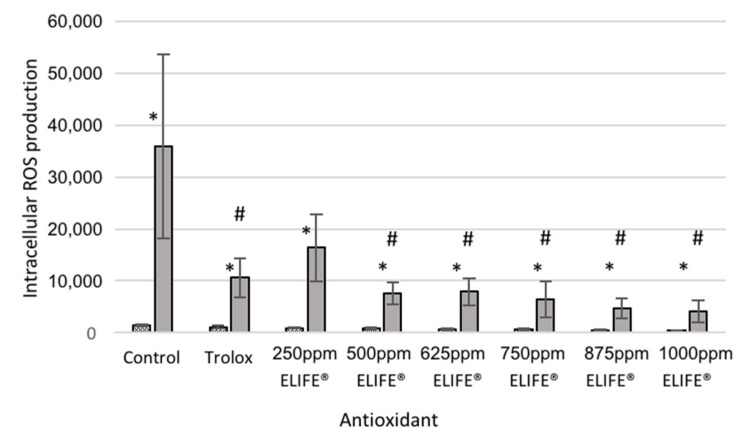
ROS production after an 18-h pre-incubation with: control (colorless growth medium containing no antioxidant), trolox (colorless growth medium containing 2 mM trolox) and different concentrations of ELIFE^®^, followed by a one-hour incubation with medium (colorless growth medium containing no stressors, dotted bars) or 200 μM menadione (in colorless growth medium, solid grey bars). ROS production in different conditions was determined by fluorescence measurement (measured in arbitrary units, AU). Different asterisks (*) indicate statistically significant differences in ROS production in non-stressed and menadione-treated cells (i.e., stressor effect). Hashtags (#) represent a statistically significant decrease in ROS production after incubation with ELIFE^®^/trolox when compared to control cells (antioxidant effect). Data represent means ± SD (*n* = 3). *p* < 0.05.

Menadione increased the intracellular ROS production in all the studied groups (control, 26-fold increase, *p* = 0.0004; trolox, 10-fold increase, *p* = 0.0008; 250 ppm ELIFE^®^, 18-fold increase, *p* = 0.0004; 500 ppm ELIFE^®^, 9-fold increase, *p* = 0.0004; 625 ppm ELIFE^®^, 11-fold increase, *p* = 0.0127; 750 ppm ELIFE^®^, 11-fold increase, *p* = 0.0025; 875 ppm ELIFE^®^, 10-fold increase, *p* = 0.0068; 1000 ppm ELIFE^®^, 11-fold increase, *p* = 0.0043) (Figure 5). To test the antioxidant capacity of trolox and ELIFE^®^, ROS production when cells were incubated with ELIFE^®^ (at different concentrations) was compared with ROS production in trolox-treated cells and with that of non-treated control cells. Fluorescence values decreased to 30%, 46%, 21%, 22%, 18%, 13% and 12% of control values when cells were pre-treated with trolox or 250 ppm, 500 ppm, 625 ppm, 750 ppm, 875 ppm and 100 ppm ELIFE^®^, respectively. Fluorescence was statistically higher in control than in all other treatments except 250 ppm ELIFE^®^ (Figure 5). Moreover, a statistically significant antioxidant effect was observed in non-stressed cells incubated with 675 and 1000 ppm ELIFE^®^; in this case, fluorescence values were decreased to 76%, 67%, 62%, 50%, 43%, 33% and 27% of control values when cells were pre-treated with trolox or 250 ppm, 500 ppm, 625 ppm, 750 ppm, 875 ppm and 1000 ppm ELIFE^®^, respectively. 

## 3. Discussion

In the present study, we have developed a medium throughput system for evaluating intracellular ROS levels in IPEC-J2 cells in cell culture microplates without the use of flow cytometry. Several aspects of the experimental procedure were optimized in order to generate oxidative damage in the cells that can be reversed by the pre-incubation with the widely-used antioxidant trolox and detected via a microplate reader. The setup is finally validated using a commercially available feed antioxidant. 

### 3.1. Imaging Plate Selection for Cell Culture

Background autofluorescence is a major concern in the bio-imaging of cells [48]. Polystyrene well plates are frequently used but have been reported to present high autofluorescence in the range of 300–400 nm [49]. We measured background fluorescence of the well plates in three different black-wall, clear-bottom plates. The well walls of all of them were made out of polystyrene, but they differed in the materials used to manufacture the bottom of the plate. As observed in Figure 2, plastic plates led to three-times higher autofluorescence than film plates, the latter showing slightly higher (1.3-fold) autofluorescence than glass plates. Even though the excitation/emission range used to detect ROS is higher, we observed a high level of autofluorescence in plastic plates, which hampers the proper quantification of the fluorescence stemming from the ROS detection probe. These results provide a clear proof that both, film and glass plates, should be considered as an alternative to plastic plates for fluorescence imaging of cells when a low autofluorescence signal is required. Although both film and glass well plates are good alternatives based on their lower autofluorescence, film plates supported better growing of the cells. The fact that the bottom is coated with a very thin (25 µm) film layer that is permeable to gases ensures an optimal oxygenation and balance with the external atmosphere [50] supporting cell growth. Therefore, film plates provide the best compromise between background signal and cell growth.

### 3.2. Intracellular Oxidative Stress

#### 3.2.1. Plate Reading Conditions

To test whether CM-H_2_DCFDA stays within the cell and leakage is negligible, we measured absorbance before and after discarding the washing buffer containing potential un-trapped or leaked CM-H_2_DCFDA. The AIC and CV analysis showed less variability when fluorescence was read before discarding the washing buffer. Previous works determining fluorescence of CM-H_2_DCFDA or similar probes also carried out the measurements in wells containing cell culture medium [19,51]. On the other hand, mean absorbance values were 10 times higher in full when compared to empty wells. In this regard, it is important to note that IPEC-J2 cells (as enterocytes), may be more efficient at transporting the probe extracellularly by active efflux. Moreover, other factors might contribute to this striking difference, namely autofluorescence of the washing buffer and/or the non-oxidized probe (which can be neglected [51]) and the permeation of ROS species to the extracellular space through the plasmatic membrane, which is a rapid (although limited) process [52]. Thus, even though the leakage of CM-H_2_DCFDA is reported to be lower than that observed in non-chloromethyl derivatives, we observed in this experiment that it is important enough to be accounted for. Measuring only intracellular CM-H_2_DCFDA fluorescence may lead to underestimation of ROS production and therefore, we recommend to quantify both intracellular and leaked CM-H_2_DCFDA. 

#### 3.2.2. Experimental Setup

To assess oxidative stress, two different setups were compared. The protocol previously used in our lab [17,18], carried out the incubation of the CM-H_2_DCFDA probe prior to exposure to the stressor agent. This protocol produced low fluorescence values that did not allow us to quantify intracellular ROS levels using a microplate reader. In the previous experiment using this protocol [17,18], intracellular ROS levels were determined via flow cytometry. This difference suggests that the faster and cost-effective protocol described in this report, entails a loss in sensitivity and specificity when compared to flow cytometry. A slight change in the chronology of the protocol (i.e., the cells were incubated with the fluorescence probe after being exposed to the stressors) increased the fluorescence signal, to a signal that was detectable and quantifiable by the plate reader. Two main aspects may contribute to a better signal in the modified protocol: first, the ROS-detection probe is more prone to cross biological membranes when it is not attached to ROS (thus, both entrance and exit to the cell occurs simultaneously in non-stressed cells). Second, in the protocol used by Vergauwen and coworkers [17,18], there is at least a two-hour lapse between the incubation with CM-H_2_DCFDA and the reading step. This could decrease the level of the hydrolyzed and oxidized probe to below the detection limits of the plate reader [53]. Similarly, a non-chloromethyl derivative of CM-H_2_DCFDA probe used in a setup with Caco-2 cells, led to a decrease in fluorescence values when incubation times increased, suggesting a loss of the probe from the cells or instability of the probe [19].

#### 3.2.3. Oxidative Stress Assessment 

Two criteria were used to select the most appropriate stressor and concentration. First, the compound must increase intracellular ROS accumulation. Second, the oxidative stress must be counteracted by incubating the cells with trolox. Two stressors, menadione and H_2_O_2_ were used at different concentrations to induce oxidative stress in IPEC-J2 cell monolayers. Three identical experiments were run for a relatively long period of time to test the reproducibility of our results. 

When including data from all three experiments, neither menadione (100 µM) nor H_2_O_2_ (1 to 2 mM) produced an increase in intracellular ROS. This lack of effect contrasts with previous literature, where the two stressors used in the present work produced an increase in oxidative stress and cell death at concentrations as low as 30 µM (menadione, [54]) and 500 µM (H_2_O_2_, [17,36]). Different aspects, such as the cell line, the growing conditions and the outcome of interest may affect the sensitivity of the culture to the presence of exogenous stressors, which makes it necessary to accord the concentrations to the specificities of the different experiments [17]. On the other hand, 2.5 and 3 mM H_2_O_2_ and 150 to 250 µM menadione produced a significant increase in intracellular ROS when compared with non-stressed cells. Among these concentrations, only 200 µM menadione consistently increased ROS production in all three experiments, whereas 150 and 250 µM menadione significantly increased intracellular ROS only in two out of three experiments. Thus, we identified 200 µM menadione as the most consistent stressor agent in our system.

Regarding the effects of the stressor on cell viability, concentrations of 100, 200 and 400 µM menadione increased cellular respiration (potentially leading to an increase in ROS accumulation) without affecting viability in cultured Molt-4 cells in a previous study [55]. In contrast, incubation of human leukemia Jurkat T-cells with 250 µM menadione increased cell apoptosis by 46% [36]. Such increase in cell death at similar concentrations has also been reported in studies involving different cell lines [40,54]. In the present study cell viability was affected in all menadione and H_2_O_2_-treated cells and therefore, we opted to consider this in the calculation of intracellular ROS levels. Although the need for standardization of ROS production as a function of the protein level has been previously stated [51], we propose a new approach based on assessing cell viability rather than protein content. This method quantifies only viable cells and can be easily and quickly performed after measuring fluorescence.

Besides identifying the stressor/concentration that complies with our first selection criterion, the effect of trolox on the stressor capacity of menadione and H_2_O_2_ was addressed. Trolox is a water-soluble antioxidant synthesized as a vitamin E derivative in 1974 [56]. It is commonly used as a standard for antioxidant capacity assays. Our data demonstrate that trolox can effectively counteract ROS accumulation caused by the exposure of cells to 2 mM and 3 mM H_2_O_2_ pooled data (*n* = 9). However, these results are not consistent when looking at individual experiments and only 2 mM was detected to be affected by a pre-incubation with trolox in Ex1. Similarly, 100 to 200 µM menadione could be reverted by trolox (pooled data, *n* = 9) but only 200 µM menadione is systematically reverted by trolox in all three experiments. The effects of menadione at 100 and 150 µM could be prevented by trolox in two and one experiments, respectively. The decrease in fluorescence after trolox pre-treatment was larger in 200 µM menadione than in any other treatment. The antioxidant capacity of trolox against H_2_O_2_ was previously addressed in porcine enterocytes and human oligodendrocytes, although the H_2_O_2_ concentrations used were smaller than those reported in the present study (1 mM and 100 µM, respectively). Moreover, trolox has been reported as an effective tool to palliate the oxidative stress induced by menadione in different setups [57,58]. 

The cell system presented in this study shows high experimental variability that was already detected during preliminary tests. This contrasts with the reported CV (<9%) in a previous study evaluating a Caco-2 cells-based system for antioxidant research [19]. On the other hand, when assessing different antioxidant indices in IPEC-J2 cells, the reported CV was as high as 59% [59], suggesting that IPEC-J2 cells-based systems are generating less reproducible results than cancerous Caco-2 cells. Comparison between different cell lines may be delicate, especially when it comes to cancerous and non-cancerous cells, as differences regarding membrane repair capacity, metabolism or cell cycle exist [60,61,62]. In addition, differences regarding antioxidants resilience are observed between cancerous human cells and non-carcinogenic, non-transformed intestinal epithelial cell lines [17]. All these differences may affect not only the response to different stimuli, but also the reproducibility of the setup. To account for this high cell variability observed during preliminary tests, we decided to repeat this experiment in three different occasions, within a period of six months. This allowed us to check whether the high variability was systematically found, which was the case. As expected, higher variation was observed when pooling data from all the experiments, probably due to greater changes in environment conditions between more distant experiments. Despite the higher variability, the large effect of 200 µM menadione as a stressor and 2 mM trolox as an antioxidant led to a consistent modulation of ROS production in our cell culture system. This could be obtained with as few as three replicates. Since the goal of this system is to provide the researcher with fast and cost-effective but accurate results, we aimed at maintaining the number of replicates as low as possible. Furthermore, in this line, we would like to stress the importance of running different replicates in different weeks to avoid pseudoreplication. This is a problem commonly found in cell culture and animal experiments that leads to biased results, lower variation and increased false positives [63]. In the system described herein, and in spite of the aforementioned high variability, 200 µM menadione and 2 mM trolox were selected as reliable stressor and antioxidant that produced consistent results across different experiments. 

#### 3.2.4. Validation of the System with ELIFE

Incubation of IPEC-J2 cells with ELIFE^®^ at concentrations between 500 and 1000 ppm produced a significant reduction in ROS production after induction of oxidative stress by 200 µM menadione when compared to non-treated cells. ELIFE^®^ includes different polyphenolic extracts in its formulation. These extracts, which boost the activity of vitamin E present in feeds, may also exert the observed antioxidant activity by means of different mechanisms of action, including radical scavenging and enhancement of the expression of antioxidant enzymes [64]. Polyphenols have been extensively studied and reported to improve the oxidative status of cells in culture [65,66] but also animals (for references see [67]) and humans [68,69]. Our setup allowed the identification of an effective antioxidant concentration of ELIFE in a simple and fast way (total ROS stimulation and assessment time is about 4.5 h, of which 1 h is hands-on). Indeed, the concentrations identified as effective in our study are very similar to the real dose used in feed (1 kg/Tm). This method proved to be a useful tool for the screening of doses of antioxidants prior to their use in further (in-vivo) experiments. 

## 4. Materials and Methods 

### 4.1. Well Plate for In Vitro Anti-Oxidant Capacity

In a preliminary trial, high autofluorescence was observed for all conditions when using polystyrene cell culture plates. We hypothesized that plate autofluorescence could be the reason behind this observation. To corroborate this and overcome the problem, prior to start the culture of IPEC-J2 cells, autofluorescence values were assessed in different cell culture plates in order to select the one with the lowest background signal. Three 96-well plates were considered: polystyrene cell culture microplate μClear^®^ (Greiner Bio-One, Volvoorde, Belgium; Cat No 655079) (Plastic), cell imaging microplate 25-μm film bottom (Eppendorf, Aarschot, Belgium Cat No 0030741013) (Film) and cell imaging microplate 170-μm coverglass bottom (Eppendorf, Aarschot, Belgium; Cat No 0030741021) (Glass). Fluorescence (Ex/Em: 495/520 nm) was measured at 21 °C using a fluorospectrophotometer (Infinite^®^ M200 PRO, Tecan, Männedorf, Switzerland) in plates containing 200 μL of DMEM/F-12 mix (Dulbecco’s modified Eagle medium, Ham’s F-12 mixture), 1.5 mM HEPES, 5% (*v*/*v*) fetal bovine serum, 1% (*v*/*v*) insulin-transferrin-selenium mixture, 1% (*v*/*v*) penicillin-streptomycin mixture and 2.5 μg/mL fungizone (Invitrogen, Merelbeke, Belgium) (growth medium). 

### 4.2. Cell Line and Culture Conditions

**Figure 6:**
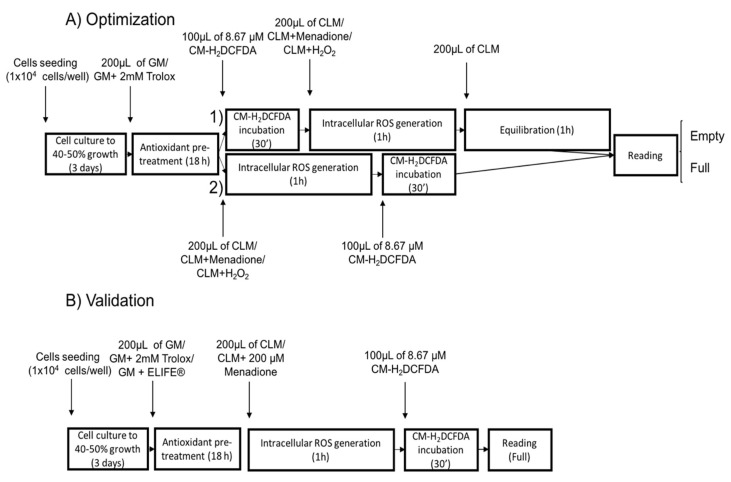
Graphical overview of the different steps included in (**A**) the optimization and (**B**) validation of the in vitro oxidative stress measurement. (1) and (2) are different protocols tested during the optimization process in order to obtain the best signal-to-noise ratio. GM refers to growth medium, CLM refers to colorless growth medium and ROS to reactive oxygen species. Fluorescence (Ex/Em: 495/520 nm) was detected under two different conditions: before (full) and after (empty) discarding the buffer containing the CM-H2DCFDA probe and washing the wells twice with PBS.

For a graphical overview of the time course of the different protocols and their respective steps, see Figure 6A. The IPEC-J2 cells (ACC 701, DSMZ, Braunschweig, Germany) were cultured in growth medium (see above) at 37 °C and 5% CO_2_ [18]. The culture medium was refreshed daily. Approximately 1 × 10^4^ cells were plated per well in a 96-well plate (25-μm flat film bottom, Eppendorf) and grown for 3 days when 40–50% confluence was attained. Cell plates were imaged under a light microscope (Model CKX 41, Olympus, Tokyo, Japan) combined with a digital color camera (Model UC 30, Olympus, Tokyo, Japan). The Olympus cellSens Standard software (Olympus, Tokyo, Japan) was used to capture the images without any further processing. Passage (passage number 4–5), number of seeded cells and growing period were standardized in order to achieve a homogeneous cell counting prior to the in vitro test. 

### 4.3. Antioxidant Pre-Treatment 

Cells were washed twice with colorless DMEM/F-12 mix without additives (washing buffer) and pre-treated (overnight, 18 h) with 200 μL of either growth medium (control group) or growth medium containing the antioxidant trolox (Sigma-Aldrich, Overijse, Belgium) (trolox group). The incubation time and concentration of trolox were established based on previous data [18]. Trolox (2 mM) was dissolved in growth medium by sonication and filtered (0.2 μm, Acrodisc Syringe Filters with HT Tuffryn Membrane, PALL, New York, NY, USA) immediately before its application to the IPEC-J2 cells.

### 4.4. Oxidative Stress Assessment

The intracellular oxidative stress was analyzed using a ROS-sensitive probe: 5-(and-6-)-chloromethyl-2′,7′-dichlorodihydrofluorescein diacetate, acetyl ester (CM-H_2_DCFDA) (Ex/Em: 492–495/517–527 nm) (Invitrogen, Merelbeke, Belgium). The probe detects intracellular H_2_O_2_ and its downstream products. Fluorescence (Ex/Em: 495/520 nm) was detected as mentioned before, under two different conditions: before (full wells) and after (empty wells) discarding the buffer containing the CM-H_2_DCFDA probe and washing the wells twice with PBS. These two measuring conditions were tested to assess the ability of the probe to stay inside the cells and/or to be oxidized by free radicals produced and secreted by the cells to the extracellular fluid. 

Positive (CM-H_2_DCFDA probe incubated with 15% (*v*/*v*) H_2_O_2_) and negative (CM-H_2_DCFDA probe incubated with growth medium with and without (blank) the stressor agent menadione in the absence of cells) controls were included in every experiment. Negative control wells indicate whether or not the probe reacts with molecules present in the washing buffer or with the stressor itself. Fluorescence values were calculated from each well after subtracting the blank signal.

Different concentrations of two oxidants (menadione and H_2_O_2_) were evaluated for their oxidative capacity, sensitivity to an anti-oxidant and test reproducibility. In a preliminary test, 100 μM menadione and 1mM H_2_O_2_ did not affect intracellular ROS levels. Therefore, these concentrations were not retained.

First, the protocol described by Vergauwen and co-workers [17,18] was followed (protocol 1 in Figure 6A). Briefly, the IPEC-J2 monolayer was pre-incubated overnight with or without trolox. The next day, the cells were washed twice, followed by incubation for 30 min with 100 μL of 8.67 μM CM-H_2_DCFDA (diluted in washing buffer) after which the cells were washed twice. Subsequently, the cells were incubated for 1 h with colorless medium containing no stressor (medium), H_2_O_2_ (H_2_O_2_) (2 or 3 mM, Sigma-Aldrich, Overijse, Belgium) or menadione sodium bisulfite (menadione) (200 or 300 μM, Sigma-Aldrich, Overijse, Belgium) followed by washing twice. Finally, the colorless culture medium was refreshed and equilibrated for 1 h before analyzing the fluorescence. 

Second, in order to obtain a better signal-to-noise ratio, the setup used in the first protocol was slightly modified (protocol 2 in Figure 6A): after pre-treatment with growth medium or trolox, cells were washed twice with washing buffer and incubated for 1 h with colorless medium containing no stressor (medium), H_2_O_2_ (H_2_O_2_) (2 or 3 mM, Sigma-Aldrich, Overijse, Belgium) or menadione sodium bisulfite (menadione) (200 or 300 μM, Sigma-Aldrich, Overijse, Belgium). After treatment with or without antioxidants and stressors, the cells were washed twice with washing buffer and incubated for 30 min with 100 μL of 8.67 μM CM-H_2_DCFDA (diluted in washing buffer). Fluorescence was measured immediately after this last step as described above. 

**Figure 7:**
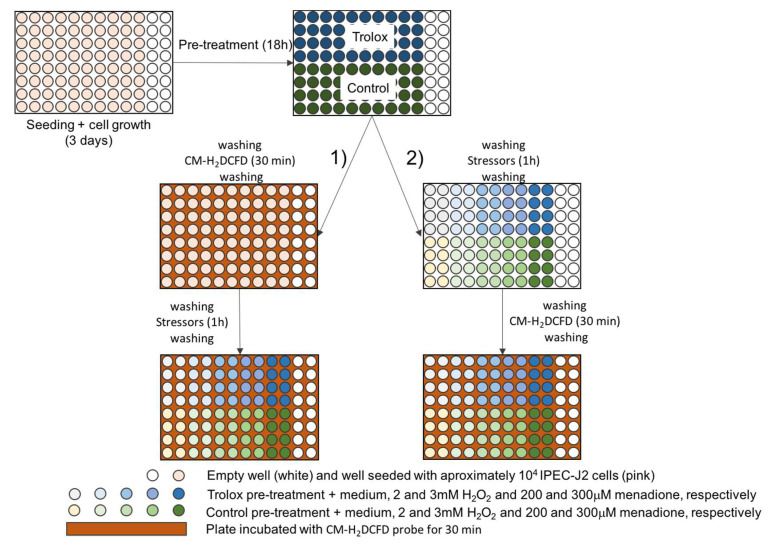
Plate layout during optimization of the in vitro oxidative stress assay. (1) and (2) are different protocols tested during the optimization process in order to obtain the best signal-to-noise ratio.

The layout of the plates can be observed in Figure 7.

### 4.5. Cell Viability

To account for differential growth between wells and for the potential increase in cell death after incubation with oxidants, a viability assay was performed in the ROS-assayed cells after fluorescence reading. The cells were washed twice and incubated in 100 μL washing buffer plus 50 μL 0.01% (*w*/*v*) neutral red dye (Janssen Chimica, Beerse, Belgium) for 2 h in the same growing conditions. Afterwards, the cells were washed twice with phosphate-buffered saline (PBS, pH 7.4, 0.01 M) to remove the neutral red dye that was not incorporated by the lysosomes of living cells. Subsequently, the dye was extracted from the cells using 100 μL of 50% (*v*/*v*) ethanol solution (in 0.05 M NaH_2_PO_4_) [18]. The absorbance was measured at 540 nm using the a fluorospectrophotometer (Infinite^®^ M200 PRO, Tecan, Männedorf, Switzerland). The absorbance value of each well was then used as a correction factor in the following manner:

$$F_{\mathrm{corr}}=\left(F_{w}-F_{\mathrm{blank}}\right)/\left({\mathrm{Abs}}_{w}-{\mathrm{Abs}}_{\mathrm{blank}}\right)$$

where F_corr_ is the corrected fluorescence value; F_w_ is the raw fluorescence value obtained from the fluorospectrophotometer for the wth well; F_blank_ is the fluorescence value for the blank well, Abs_w_ is the absorbance value obtained from the fluorospectrophotometer for the wth well and Abs_blank_ is the absorbance value for the blank well after viability was assessed. 

### 4.6. Validation of the System with ELIFE

The optimized in vitro model was validated with a commercially available in vivo antioxidant, i.e., ELIFE^®^ (Impextraco, Heist-op-den-Berg, Belgium) and compared with trolox. Cell culture conditions were as described in Section 2.2. Antioxidant pre-treatment with growth medium, trolox and ELIFE was performed as detailed in Section 4.3. Based on dose data from the manufacturing company, ELIFE was included at different concentrations, including 250, 500, 625, 750, 875 and 1000 ppm. After the cells were pre-treated with the different concentrations, they were stressed for 1 h with menadione at 200 ppm (see results section for the optimal protocol selection), followed by incubation with the ROS-sensitive probe for 30 min and fluorescence reading, as stated in Section 4.4 (Figure 6B). This experiment was repeated three times in three consecutive weeks.

### 4.7. Statistical Analysis

Reported values are fluorescence values expressed in arbitrary units, indicative of intracellular ROS accumulation. For optimization of the experimental conditions, each experiment consisted of four technical replicates (wells) per condition and three independent replicates (the experiment was repeated three times in three consecutive weeks, using cells coming from different vials kept at −196 °C). The experiments were repeated three times, with about one month in between experiments. Data are expressed as means ± standard deviation (SD) (*n* = 3, where *n* refers to the number of replicates in time). Data were analyzed using JMP^®^ Pro 13 (SAS Institute Inc., Addison, TX, USA). Data were log-transformed to meet normality and homoscedasticity assumptions. A mixed model was used to analyze corrected fluorescence data in each individual experiment. First, the stressor effect was analyzed in control (i.e., no antioxidant pre-treatment) cells. The stressor agent (medium, 2 mM and 3 mM H_2_O_2_, 200 μM and 300 μM menadione) was included as the fixed effect and the plate, nested within stressor, as a random effect (this is equivalent to a one-way ANOVA after averaging the results from the same plate). The post-hoc Tuckey test was used to discriminate differences between means. Then, we evaluated whether the antioxidant pre-treatment (control vs. trolox) could revert the effects of the different stressor agents. To do so, different mixed models were fitted for the different stressor agents where the antioxidant was included as the fixed effect and the plate, nested within antioxidant, as the random effect. Besides analyzing each experiment separately, the data from the three different experiments run in the course of six months were pooled and mixed models were fitted. In this case, the average of the four technical replicates were calculated. Fixed factors included the stressor agent or the antioxidant, depending on the effect being tested, while the experiment was included as a random factor to correct for the lesser independency expected in observations taken in the same vs. different experiments. 

Similar models were fitted to the outcome of the validation experiment. First, the effect of the stressor (200 μM menadione) was evaluated to confirm the induction of intracellular ROS and thus, the validity of the system. Second, to evaluate whether the antioxidants pre-treatment (control, trolox, and ELIFE^®^ at different concentrations) could revert the effects of menadione, different mixed models were fitted for the different antioxidants where the antioxidant was included as the fixed effect and the plate, nested within stressor, as the random effect. Dunnett test was used to identify compounds (and doses) which produced a decrease in intracellular ROS compared to control cells.

A difference between two values was considered to be statistically significant if the *p*-value was < 0.05.

## 5. Conclusions

The model presented in this study represents a fast, simple and cost-effective alternative for in vitro screening of antioxidants than can potentially be used as feed additives. To overcome the lower sensitivity and specificity attained by the proposed fluorospectrophotometer device compared to flow cytometry, we propose the use of 200 μM menadione as the stressor agent and a cell viability-based standardization to evaluate the antioxidant capacity of compounds in this in vitro model of porcine intestinal epithelial cells.

## Supplementary Materials

The following are available online at https://www.mdpi.com/1422-0067/21/19/7263/s1.
